# Inflammatory neuronal loss in the substantia nigra induced by systemic lipopolysaccharide is prevented by knockout of the P2Y_6_ receptor in mice

**DOI:** 10.1186/s12974-021-02280-2

**Published:** 2021-10-11

**Authors:** Stefan Milde, Francesca W. van Tartwijk, Anna Vilalta, Tamara C. Hornik, Jacob M. Dundee, Mar Puigdellívol, Guy C. Brown

**Affiliations:** Department of Biochemistry, University of Cambridge, Cambridge, UK

**Keywords:** Microglia, Phagocytosis, Neuroinflammation, Cell death, Parkinson’s disease, Neurodegeneration, P2Y_6_R, Phagoptosis

## Abstract

Inflammation may contribute to multiple brain pathologies. One cause of inflammation is lipopolysaccharide/endotoxin (LPS), the levels of which are elevated in blood and/or brain during bacterial infections, gut dysfunction and neurodegenerative diseases, such as Parkinson’s disease. How inflammation causes neuronal loss is unclear, but one potential mechanism is microglial phagocytosis of neurons, which is dependent on the microglial P2Y_6_ receptor. We investigated here whether the P2Y_6_ receptor was required for inflammatory neuronal loss. Intraperitoneal injection of LPS on 4 successive days resulted in specific loss of dopaminergic neurons (measured as cells staining with tyrosine hydroxylase or NeuN) in the *substantia nigra* of wild-type mice, but no neuronal loss in cortex or hippocampus. This supports the hypothesis that neuronal loss in Parkinson’s disease may be driven by peripheral LPS. By contrast, there was no LPS-induced neuronal loss in P2Y_6_ receptor knockout mice. In vitro, LPS-induced microglial phagocytosis of cells was prevented by inhibition of the P2Y_6_ receptor, and LPS-induced neuronal loss was reduced in mixed glial–neuronal cultures from P2Y_6_ receptor knockout mice. This supports the hypothesis that microglial phagocytosis contributes to inflammatory neuronal loss, and can be prevented by blocking the P2Y_6_ receptor, suggesting that P2Y_6_ receptor antagonists might be used to prevent inflammatory neuronal loss in Parkinson’s disease and other brain pathologies involving inflammatory neuronal loss.

## Background

Inflammation contributes to the pathology and neuronal loss of various CNS diseases [1–4], but the mechanisms are unclear, and thus means to prevent inflammatory neuronal loss are underdeveloped. The inflammatory response of the brain is mainly mediated by microglia, which are resident brain macrophages that when inflamed can kill neurons by multiple mechanisms, including by phagocytosing neurons [1–3]. Dead neurons do not accumulate significantly in neurodegenerative diseases, and one potential cause of this is that neurons are phagocytosed when alive [5, 6]. Phagocytosis of live cells normally results in death of the engulfed cells, a type of cell death termed phagoptosis, i.e., cell death by phagocytosis [7]. We have shown that activated microglia become highly phagocytic and cause neuronal loss by phagocytosis of stressed-but-viable neurons [8–11].

One cause of inflammation is lipopolysaccharide/endotoxin (LPS), derived from the cell wall of gram-negative bacteria, which activates inflammation via Toll-like receptor 4 (TLR4) on immune cells, such as microglia [12]. A variety of conditions, such as liver cirrhosis, gingivitis, sepsis and Alzheimer’s disease elevate plasma LPS levels, and eventually cause neurodegeneration [12]. One cause of elevated LPS is infection with gram-negative bacteria, and bacterial infections are common in patients with neurodegenerative diseases and can both exacerbate clinical symptoms and accelerate disease progression [13, 14]. Recently, it was reported that plasma LPS levels were substantially elevated in a subset of Parkinson’s disease patients, particularly those at high risk of dementia [15]. Gut dysfunction is one of the earliest symptoms of Parkinson’s disease, and may result in translocation of LPS from the gut (where levels are very high) into the blood (where levels are normally very low) [12, 15, 16]. Addition of LPS (at levels found in disease) to the blood of healthy volunteers results in rapid body and brain inflammation with microglial activation [17]. This all supports the hypothesis that LPS and microglial activation contribute to neurodegeneration [12], but further evidence and mechanisms are required before potential treatments can be devised.

Microglial phagocytosis of neurons requires the P2Y_6_ receptor (P2Y_6_R), encoded by the *P2ry6* gene, and activated by extracellular uridine diphosphate (UDP) [18, 19]. In the brain, P2Y_6_R is almost exclusively expressed by microglia [18, 20, 21]. The human *P2RY6* gene is highly homologous to the mouse gene [22, 23], and GWAS data indicate that variants of the human gene are associated with cognitive performance [24], consistent with a role in brain development or neuroprotection. *P2ry6* gene expression levels were elevated in monocytes from Parkinson’s disease patients, and expression was increased in a microglial cell line treated with LPS [25]. Microglial P2Y_6_R has been found to mediate microglial phagocytosis of kainite-treated neurons, which released UTP/UDP that activated P2Y_6_R on microglia, inducing engulfment of these neurons [18]. However, in that work, it was assumed that the neurons releasing UDP were dying, and it was not determined whether inhibiting P2Y_6_R prevented neuronal loss [18]. We found that an inhibitor of P2Y_6_R (MRS2578) prevented neuronal loss induced by LPS injected into brain or cell cultures [9], but we did not test whether: (i) this was mediated by P2Y_6_R, and (ii) lack of P2Y_6_R was protective against peripheral LPS. The work described here seeks to determine whether the inflammatory neuronal loss induced by peripheral LPS can be prevented by blocking the microglial P2Y_6_ receptor. The work shows that peripheral LPS specifically induces neuronal loss in the *substantia nigra*, and this neuronal loss is fully prevented by *P2ry6* knockout. This suggests that microglial phagocytosis contributes to inflammatory neuronal loss, and that blocking P2Y_6_R might be neuroprotective in Parkinson’s disease.

## Methods

### Animals

*P2ry6* knockout (*P2ry6*^*−/−*^) mice were kindly provided by Bernard Robaye (ULB Brussels) and maintained on a C57Bl/6 background (Charles River Laboratories). *P2ry6*^*−/−*^ mice and wild-type (WT) littermates were used to establish homozygous WT and *P2ry6*^*−/−*^ sub-lines. In offspring from these sub-lines, littermates were randomly assigned to control and LPS treatment groups. Details of experimental animals used for each study are given in Table 1.

**Table 1:** Details of experimental animals used

Study	Treatment group	Genotype	Number of animals	Sex	Age range (weeks)	Weight range at start of procedure (g)
I.p. injection of LPS (group 1)	Control	WT	4	Male	17–18	21–28
	Treatment	WT	4	Male	17–18	20–25
	Control	*P2ry6*^*−/−*^	4	Male	18–20	22–29
	Treatment	*P2ry6*^*−/−*^	4	Male	18–20	20–26
I.p. injection of LPS (group 2)	Control	WT	4	Male	20–26	32–36
	Treatment	WT	4	Male	20–26	34–38
	Control	*P2ry6*^*−/−*^	4	Male	20–25	28–35
	Treatment	*P2ry6*^*−/−*^	4	Male	20–25	29–35

### Intraperitoneal injection of LPS

LPS from *Salmonella abortus equi* (S-form; Enzo Life Sciences) or PBS (control) was injected intraperitoneally at a dose of 1 µg/g/day in a total volume of 100 µl at the same time of day on 4 subsequent days. Animals were sacrificed 14 days after the final LPS injection.

### Transcardial perfusion and tissue sectioning

Mice were given terminal anaesthesia (150 µl Euthatal i.p.) and, once unresponsive to pain, perfused transcardially, through a 25-gauge needle, with 20 ml PBS, pH 7.4 followed by 60 ml 4% PFA, pH 7.4 using a perfusion pump with flow rate of 4 ml/min. Following perfusion, brains were removed and post-fixed overnight in the same solution, and cryoprotected by immersion in 10–30% sucrose solution until sectioning. Brain sections were cut to 20 µm thickness using a Compresstome VF-200 vibratome (Precisionary Instruments), collected on Superfrost Plus slides (Thermo Fisher) and dried overnight. Serial coronal sections (25 µm) through the whole brain were collected using a sliding microtome and placed in PBS as free-floating sections.

### Immunostaining of brain slices

All steps were carried out at room temperature unless indicated otherwise. Brain slices were re-hydrated for 1 h in PBS and heat-mediated antigen retrieval was carried out at 95 °C for 20 min in citrate buffer (10 mM sodium citrate, 0.05% Tween 20, pH 6.0). Following washes in PBS (6 × 10 min), slices were permeabilized in PBS with 0.5% Triton X-100 for 10 min followed by 1 h incubation in blocking solution (20% normal goat serum in PBS). Slices were then incubated in primary antibody solution (5% normal goat serum in PBS plus appropriate primary antibody) at 4 °C overnight. Following washes in PBS (6 × 10 min), slices were incubated with secondary antibody for 2 h, washed (6 × 10 min, PBS) and mounted using Vectashield mounting medium with DAPI (Vector Laboratories). Primary antibodies used were Anti-NeuN (Millipore, mouse monoclonal, 1:500 dilution), anti-IbaI (Wako, rabbit polyclonal, 1:500 dilution) and anti-Tyrosine Hydroxylase (Merck, rabbit polyclonal, 1:500 dilution). Secondary antibodies were Alexa-Fluor 488 anti-mouse, Alexa-Fluor 568 anti-rabbit and Alexa-Fluor 633 anti-rabbit (all ThermoFisher, goat, 1:1000 dilution). Imaging was carried out on an Olympus FV1000 upright laser-scanning confocal microscope with a 60×, 1.35 NA oil immersion objective using 488, 559 and 635 nm laser lines.

### Image analysis using ImageJ 1.49 software

All image analysis was carried out using ImageJ 1.49 software. All manual counting and quantification was performed blinded to genotype and treatment condition. Neuronal numbers were quantified for the CA1 and CA3 regions of the hippocampus as well as the outer layer of the motor cortex. Neuronal density within these regions was quantified by counting of neuronal cell bodies within areas of fixed size. CA1 and CA3 regions were ‘straightened’ in Fiji [26], allowing rectangles of constant size to be placed at the same position relative to identifiable brain structures. CA1 and CA3 width was determined at three fixed points along their length using line intensity profiles of the NeuN stain. Four brain sections were analyzed per animal, with both right and left sides of the hippocampus included in the analysis. For quantification of neuronal density in motor cortex, regions of interest of fixed size were placed randomly in anatomically matched sections and NeuN^+^ cells counted manually. Three areas were counted on each side of the midline for a total of four sections per animal. For quantification of cell densities (neuron and microglia) in SN, left and right sides of four anatomically matched sections were analyzed for each animal. Dopaminergic cells were counted as tyrosine-hydroxylase-positive cells in the entire *substantia nigra pars compacta* (SNpc). For the same area, NeuN^+^ neurons and Iba1^+^ microglia were counted and cell counts were normalized to the area.

### Cell line experiments

BV-2 are a murine microglial cell line, immortalised by a v-raf/v-myc-carrying retrovirus [27]. BV-2 (passage < 30) were maintained in DMEM supplemented with 10% FBS (glial medium). At confluence, cells were harvested using 0.5% trypsin in PBS, seeded at 4 × 10^4^ cells/well (microscopy) or 5 × 10^4^ cells/well (flow cytometry) in DMEM supplemented with 0.5% FBS (0.5% glial medium), and left to adhere for 24 h prior to treatment.

PC12 are a rat pheochromocytoma cell line [28], that can be differentiated into dopaminergic neuron-like cells [29]. PC12 were maintained in RPMI medium supplemented with 10% horse serum and 5% FBS (PC12 medium). To generate differentiated PC12, cells were seeded on collagen (4 μg/well) at 5 × 10^4^ cells/well in RPMI supplemented with 0.5% horse serum and 100 ng/ml NGF, and left to differentiate for 3 days.

BV-2 cells were seeded at 1.25 × 10^4^ cells/well on TAMRA-stained (50 µM) differentiated PC12 cells. Mixed cultures were stimulated with LPS (100 ng/ml) after 24 h, and MRS2578 (1 µM) was added daily where indicated. After a further 72 h, cultures were stained with Hoechst 33342, propidium iodide and IB_4_ and imaged. The number of TAMRA-positive BV-2 cells (BV-2 having phagocytosed PC12) was quantified.

### Cytokine and chemokine release from microglia

Primary microglia were isolated from mixed glial cultures from wild-type and P2ry6^−/−^ mice, and treated with ± 100 ng/ml lipopolysaccharide for 16 h, then the extracellular cell supernatant was centrifuged at 10,000 RCF to remove cellular debris. Supernatant was then assayed using an ELISA for 62 mouse cytokines and chemokines as per the manufacturer’s instructions (abcam, ab133995). Densitometric measurements were quantified using ImageJ, whereby intensity values were normalised between membranes using positive control spots. The fold change ± LPS for P2ry6^+/+^ and P2ry6^−/−^ microglia was tested for significance using unpaired *t* tests for each cytokine/chemokine, followed by a Holm–Šídák multiple comparisons test. There were no significant differences for any cytokine/chemokine: VEGF, VCAM-1, thrombopoietin, sTNF RI, sTNF RII, TNF α, TIMP-1, TECK, TCA-3, TARC, SDF-1 α, SCF, RANTES, P-selectin, PF-4, MIP-3 α, MIP-3 β, MIP-2, MIP-1 γ, MIP-1 α, MIG, M-CSF, MCP-5, MCP1, lymphotactin, L-selectin, LIX, leptin, leptin R, KC, IL-17, IL-13, IL-12 p70, IL-12 p40/70, IL-10, IL-9, IL-6, IL-4, IL-3 Rb, IL-3, IL-2, IL-1β, IL-1α, IGFBP-6, IGFBP-5, IGFBP-3, IFN-γ, GM-CSF, GCSF, fractalkine, Fas ligand, eotaxin-2, eotaxin, CXCL16, CTACK, CRG-2, CD40, CD30 T, CD30 L, BLC and Axl (based on three independent experiments).

### Primary cell culture experiments

Primary mixed neuronal/glial cultures were prepared from cerebella of postnatal days 3–5 WT and *P2ry6*^*−/−*^ mouse pups as previously described [30]. Cells were treated with 500 ng/ml of lipopolysaccharide for 3 days. To assess cell viability after LPS treatment, we measured the rate of reduction of MTT (3-(4,5-dimethylthiazol-2-yl)-2,5-diphenyltetrazolium) to formazan by the cells. Thus, mixed neuronal/glial cultures were incubated with MTT (0.58 mg/ml) for 2 h at 37 °C. Afterward, the converted dye was liberated from the cells and solubilized by addition of dimethyl sulfoxide (DMSO), and the absorbance intensity of *λ* = 590 nm light was measured.

### Statistical analysis

All statistical analysis was carried out using GraphPad Prism 6 and the statistical tests are indicated in the figure legends.

## Results

**Fig. 1:**
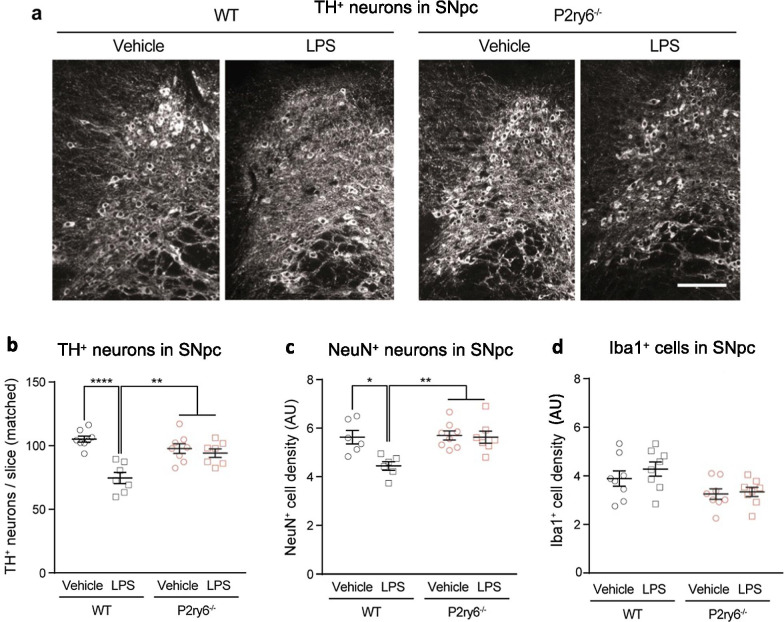
*P2ry6* knockout mice are protected against peripheral LPS-induced neuronal loss. WT and *P2ry6*^*−/−*^ mice were injected i.p. with LPS on 4 successive days and 14 days later the *substantia nigra* was sectioned and stained to quantify neuronal and microglial density. **a** Representative images of anatomically matched, anti-tyrosine hydroxylase (TH) stained sections of *substantia nigra pars compacta* (SNpc) in LPS-treated and vehicle-treated WT and *P2ry6*^*−/−*^ mice. Scale bar: 100 µm. **b** TH-positive dopaminergic neurons per section in SNpc. **c** Relative densities of NeuN-positive neurons in SNpc. **c** Relative densities of Iba-positive microglia (or other macrophages) in SNpc. Each data point represents one animal and error bars represent mean ± SEM. Statistical analysis was performed using two-way ANOVA with post hoc Tukey’s multiple comparison test. *p < 0.05, **p < 0.01, ****p < 0.0001

We tested whether P2Y_6_R knockout (*P2ry6*^−/−^) protected against the neuronal loss induced by peripheral inflammation. Mice received doses of 1 µg/g LPS intraperitoneally (i.p.) on 4 successive days, a treatment which has been shown to result in significant loss of neurons in the *substantia nigra* [31]. As expected, LPS-treated wild-type animals showed a significant reduction in the total number of tyrosine hydroxylase (TH)-positive dopaminergic neurons in the *substantia nigra pars compacta* (SNpc) (Fig. 1a, b). The total number of NeuN-positive neurons in this area was also significantly reduced in LPS-treated wild-type (WT) animals compared to vehicle-treated controls (Fig. 1c). However, the proportion of NeuN^+^ neurons lost (21%) was less than the proportion of dopaminergic neurons lost (29%), suggesting that dopaminergic neurons were somewhat more vulnerable to peripheral LPS treatment.

**Fig. 2:**
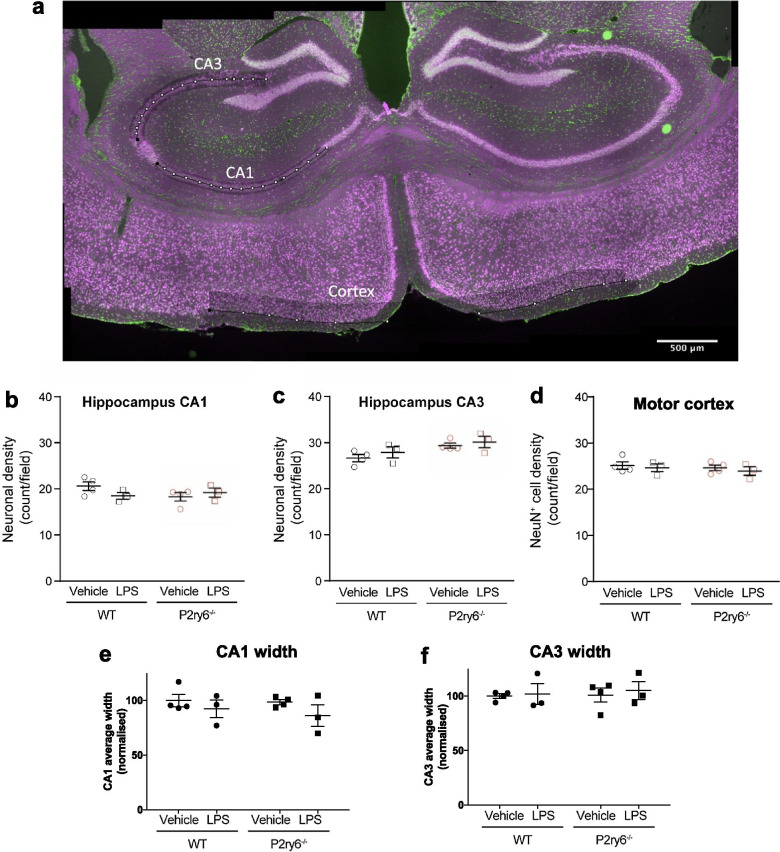
Peripheral LPS treatment does not alter neuronal densities in hippocampus or prefrontal cortex. **a** Mouse brain slice stained for nuclei (with DAPI, green) and for neuronal nuclei (with anti-NeuN antibodies, magenta). Regions of interest for quantification in CA1, CA3 and cortex are indicated (scale-bar 500 μm). NeuN^+^ neuronal densities in anatomically matched sections of **b** CA1 hippocampus, **c** CA3 hippocampus or **d** motor cortex of wild-type (WT) and *P2ry6*^*−/−*^ mice following i.p. injection of PBS (vehicle) or LPS. Mean width of **e** CA1 hippocampus and **f** CA3 hippocampus of same mice. Each data point represents one animal and error bars represent mean ± SEM. Statistical analysis was performed using two-way ANOVA, followed by Sidak-adjusted post-hoc tests, but there were no statistically significant differences

In contrast to wild-type mice, *P2ry6*^−/−^ mice had no significant LPS-induced neuronal loss, measured by tyrosine hydroxylase or NeuN (Fig. 1b, c). Thus, genetic ablation of P2Y_6_R completely prevents neuronal loss in this inflammatory model that is particularly relevant to PD [12, 32]. Neither genotype nor LPS had any significant effect on microglial density in the *substantia nigra* (Fig. 2d), indicating that P2Y_6_R ablation did not prevent neuronal loss by preventing microglial proliferation, (which is also one measure of microglia activation).

LPS treatment did not cause any change in the density of NeuN-positive neurons in hippocampal areas CA1 and CA3 or motor cortex (Fig. 2a–d), or any change in the width of CA1 or CA3 (Fig. 2e, f). This indicates selective vulnerability (or exposure) of the SNpc to peripheral LPS treatment, consistent with the hypothesis that elevated peripheral LPS may contribute to neuronal loss in Parkinson’s disease [12].

**Fig. 3:**
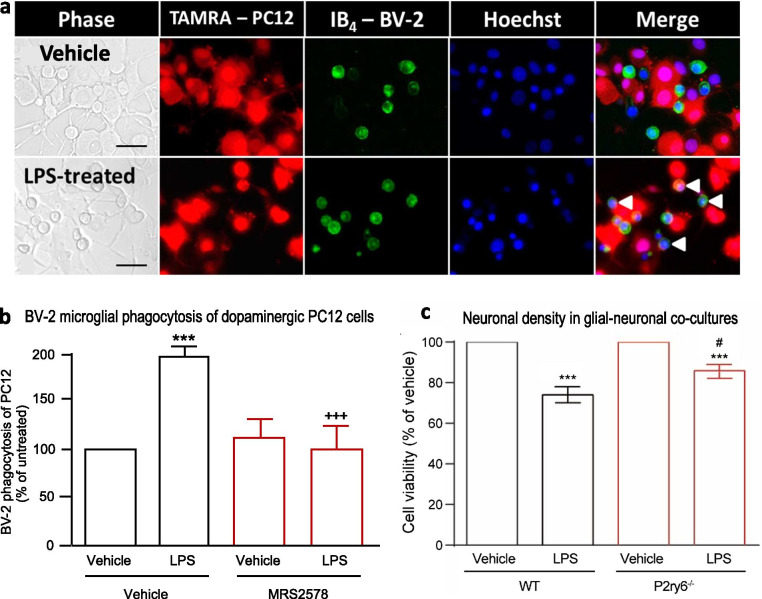
P2Y_6_R inhibition or knockout prevents LPS-induced phagocytosis and neuronal loss in culture. **a** Bright field and fluorescent images of differentiated TAMRA-stained PC12 (red), co-cultured for 3 days with IB_4_-labelled BV-2 (green), nuclei stained with Hoechst 33342 (blue) ± LPS. BV-2 phagocytosis of PC12 is visible as punctate TAMRA staining in IB_4_-stained cells (white arrowheads). Representative images; scale bar = 50 µm. **b** Quantification of BV-2 phagocytosis of PC12 cells ± LPS ± MRS2578 after 3 days. **c** Mixed neuronal–glial cultures from cerebella of wild-type (WT) or *P2ry6*^*−/−*^ mice were treated for 3 days with ± 500 ng/ml LPS, then cell viability was measured by MTT assay. Data represent mean ± SEM of at least three independent experiments. Statistical analysis was performed using one-way ANOVA with post hoc Tukey’s multiple comparison test. ***p < 0.001 compared to vehicle-treated control (first bar). ^+++^*p* < 0.001 compared to LPS-treated vehicle control (second bar). ^#^*p* < 0.05 compared to LPS-treated wild-type condition (second bar)

To help elucidate mechanisms in vitro, we co-cultured BV-2 microglia with differentiated PC12 dopaminergic neurons [33], and found that LPS induced microglial phagocytosis of neurons, which was prevented by a specific inhibitor of P2Y_6_R (1 μM MRS278) (Fig. 3a, b). This supports the ideas that LPS can increase microglial phagocytosis of neurons, and that inhibition of P2Y_6_R can prevent this and, therefore, might have therapeutic potential.

To test whether P2ry6 knockout affects the LPS-induced activation of microglia, primary microglia were isolated from wild-type and P2ry6^−/−^ mice, and treated ± LPS for 16 h, then cytokine release into the culture medium was measured using an ELISA for 62 mouse cytokines and chemokines. However, there were no significant differences between wild-type and P2ry6^−/−^ microglia in the fold change in cytokine/chemokine release induced by LPS (see “Methods”).

We have previously shown that LPS induces neuronal loss in primary glial–neuronal cultures via microglial phagocytosis [8–11], so we tested here whether this neuronal loss required the engulfment receptor P2Y_6_R. We found that addition of LPS caused a loss of viability in cultures from wild-type (WT) mice, but this was reduced in cultures from P2Y_6_R knockout (*P2ry6*^*−/−*^) mice (Fig. 3c). Thus, P2Y_6_R knockout is neuroprotective against LPS both in vitro and in vivo.

## Discussion

We tested whether *P2ry6* knockout affected LPS-induced neuronal loss in vivo and in vitro. We used a chronic peripheral LPS (intraperitoneal injection) model that has previously been described to induce dopaminergic neuronal loss in the *substantia nigra*, as indicated by loss of cells staining with TH, NeuN and/or MAP2 [31, 34]. The LPS also induced inflammatory activation of the microglia (for the first few days after LPS), including particularly the expression of phagocytic genes, such as complement C3, and the neuronal loss was prevented by knockout of C3 [31, 34, 35]. This model may be directly relevant to human PD, as intestinal permeability increases early in PD, potentially allowing leakage of bacterial products, such as LPS into the blood and peritoneum, and blood LPS levels increase in some PD patients [12, 13, 15, 36]. One source of this LPS is the gut microbiome, which is disturbed in PD and PD models, and may affect PD progression [36, 37].

We observed robust loss of dopaminergic neurons in the *substantia nigra* in wild-type mice treated with LPS; however, *P2ry6* knockout mice were protected against this neuronal loss. Interestingly, in wild-type mice, we found no evidence for LPS-induced neuronal loss in the cortex or the hippocampus of wild-type and *P2ry6* knockout mice. Thus, this peripheral inflammation-driven model of dopaminergic neuronal loss in the *substantia nigra* appears to model the selective vulnerability of this neuronal population observed in PD, and lack of P2Y_6_R protects against this neuronal loss. Consistent with this, we previously reported that the P2Y_6_R inhibitor MRS2578 prevented neuronal loss in the striatum of rats injected with LPS [9]. Similarly, Oliveira-Giacomelli et al. [38] have recently reported that MRS2578 prevented dopaminergic neuronal loss in the *substantia nigra* of rats injected with 6-hydroxydopamine, a model of PD, indicating the potential of P2Y_6_R inhibitors to reduce neuronal loss in PD. Furthermore, Yang et al. [25] reported that LPS increased P2Y_6_R expression by microglia, and that P2Y_6_R expression was increased in peripheral blood monocytes from PD patients relative to healthy controls, indicating elevated P2Y_6_R expression in PD patients, possibly due to elevated LPS, and potentially inducing P2Y_6_R-mediated phagocytosis.

We measured loss of neurons in the SNpc using tyrosine hydroxylase (TH) as a marker of dopaminergic neurons, and “neuronal nuclei” (NeuN) as a marker of total neurons. However, Cannon and Greenamyre [39] reported that a proportion of TH^+^ cells in the SNpc did not express detectable NeuN, and Ünal-Çevik et al. [40] reported that some cortical neurons lost detectable NeuN hours after brain ischaemia but had intact nuclei, implying that live neurons could lose NeuN staining. However, they attributed this to caspase-3 activation in these cells, and translocation of NeuN to the cytoplasm; thus, they concluded that counting NeuN^+^ cells could still be used to measure neuronal loss/death. TH expression can also vary in neurons [41] and can fall in Parkinson’s disease [42], but there are very few reports of neurons losing detecting TH staining while remaining live neurons, and as TH is required for dopamine synthesis, cells without TH can not be dopaminergic neurons [41]. Our finding that both TH^+^ and NeuN^+^ cells in the SNpc are reduced by LPS and this is prevented by P2Y_6_R knockout, supports the conclusion that the reduction in neuronal numbers is due to cell loss mediated by P2Y_6_R, but we cannot rule out that some of the neurons dedifferentiated and this was prevented by P2Y_6_R knockout. Similarly, as we did not directly measure microglial phagocytosis of neurons in vivo, we cannot be sure that the neuronal loss was due to microglial phagocytosis. However, this interpretation would be consistent with the mechanism of LPS-induced neuronal loss previously characterized in vitro and in vivo, and consistent with the P2Y_6_R being required for microglial phagocytosis of neurons [8, 9, 18].

In vitro, LPS induced BV-2 microglial phagocytosis of dopaminergic PC12 cells that was prevented by inhibition of P2Y_6_R, consistent with our previous findings [33]. *P2ry6* knockout protected against neuronal loss induced by LPS in primary glial–neuronal cultures. We have previously shown that this neuronal loss induced by LPS is mediated by microglial phagocytosis of stressed-but-viable neurons [8–11], and the results here are consistent with this being mediated by the engulfment receptor P2Y_6_R.

Overall, our findings indicate that the P2Y_6_ receptor contributes to neuronal loss under inflammatory conditions. This suggests that a P2Y_6_R antagonist might prevent neuronal loss in Parkinson’s and other diseases with inflammatory neuronal loss.

## Data Availability

The data sets supporting the conclusions of this article are included within the article.

## Abbreviations

CA1: Cornu Ammonis subfield 1
CA3: Cornu Ammonis subfield 3
i.p.: Intraperitoneal
ko: Knockout
LPS: Lipopolysaccharide
P2Y_6_R: Pyrimidinergic receptor P2Y_6_
SNpc: *Substantia nigra pars compacta*
UDP: Uridine diphosphate
wt: Wild-type
